# Selecting lncRNAs in gastric cancer cells for directed therapy with bioactive peptides and chemotherapy drugs

**DOI:** 10.18632/oncotarget.20977

**Published:** 2017-09-18

**Authors:** Wenyan Han, Rui Xiao, Chuanling Zhang, Qimuge Suyila, Xian Li, Xiulan Su

**Affiliations:** ^1^ Clinical Medical Research Center of The Affiliated Hospital, Inner Mongolia Medical University, Hohhot 010050, Inner Mongolia Autonomous Region, P.R. China; ^2^ Inner Mongolia Key Laboratory of Molecular Pathology, Inner Mongolia Medical University, Huhhot 010059, Inner Mongolia Autonomous Region, P.R. China; ^3^ Department of Pharmacy, Inner Mongolia Medical University, Huhhot 010110, Inner Mongolia, P.R. China

**Keywords:** gastric cancer, anti-cancer bioactive peptide, lncRNAs, profile

## Abstract

Selecting lncRNAs for directed therapy with bioactive peptides and chemotherapy drugs may be an effective approach to treating gastric cancer (GC). We show genome-scale identification and characterization of differentially expressed lncRNAs in GC cells treated with a novel anti-cancer bioactive peptide (ACBP) and the chemotherapy drug oxaliplatin (ASLB). A total of 17,897 lncRNAs were identified through pairwise comparison, including 2,074 novel lncRNAs. Of those, 1,386 lncRNAs were differentially expressed (over 1.5-fold change vs. control, *q-*value < 0.05) in response to ACBP and ASLB treatment. These included 914 upregulated and 472 downregulated lincRNAs. Functional annotation of these lncRNAs through Kyoto Encyclopedia of Genes and Genome (KEGG) pathway analysis revealed they activate metabolic pathways and protein-binding processes. Moreover, suppression of the DNA replication process and upregulation of AMP-activated protein kinase (AMPK) signaling in MKN45 cells exposed to ACBP alone or in combination with ASLB was predicted by hierarchical clustering analysis. By providing new insight into the transcriptomic effects of ACBP and ASLB in GC cells, these results provide the first evidence of ACBP inhibition of lincRNAs and may provide new mechanisms of action for ACBP and ASLB.

## INTRODUCTION

Gastric cancer (GC) is one of the major causes of cancer deaths around the world. In China, the annual mortality of GC is estimated to be as high as 16 per 100,000 population, with GC the leading cause of death among malignant tumors [1]. The incidence and mortality of GC have decreased over the past 50 years with improvement in surgical methods and chemotherapeutic treatments. However, GC remains a leading cause of death, a major economic burden, and a severe hindrance to patient quality of life.

Most patients with advanced and metastatic GC are treated with chemotherapy. Oxaliplatin (ASLB), a third-generation organoplatinum compound, induces antitumor activity by cross-strand binding of DNA as well as by blocking DNA synthesis [2]. Cell growth inhibitory effects from ASLB treatment were observed in many cancer cell lines and tumors, including those that are primarily resistant to cisplatin (CDDP) and carboplatin [3, 4]. In the treatment of GC patients, ASLB is frequently used because of its lack of nephrotoxicity or its low drug-induced ototoxicity [5]. However, the side effects of ASLB chemotherapy for GC often result in poor quality of life.

Studies have demonstrated that certain natural bioactive compounds found in food, herbs, and animals can stimulate the expression of tumor suppressor genes [6, 7]. Approximately 80% of approved chemotherapeutic agents and their sources are derived from natural compounds [7]. Thus, promoting anti-cancer activity and suppressing side effects by use of biologically active materials, including bioactive peptides, are ideal approaches to cancer prevention and potentially to anti-cancer therapy.

Anticancer bioactive peptide (ACBP), a novel antitumor agent isolated from goat liver immunized with human GC extract in our lab, shows significant and effective inhibition of tumor cell proliferation in GC, leukemia, nasopharyngeal cancer, and gallbladder cancer [8–11]. ACBP was identified as a mixture of several polypeptides with a molecular weight of approximately 8 kDa, including ubiquitin proteases and fatty acid binding protein. In addition, short-term and long-term toxicological tests in mice and rats showed ACBP had no measurable toxicities or side effects that interfere with normal physiological functions and enzyme metabolism activities.

The combination of ACBP and chemotherapeutic activity in GC cells was analyzed in the literature. The chemotherapeutic agents included 5-FU [12], ASLB [13], and cisplatin [14]. The MKN45 cells used in our study are a tumorigenic GC cell line and display the properties of cancer stem-like cells, such as self-renewal and proliferating capacity. In addition, MKN45 cells demonstrated properties of chemo-resistance and radio-resistance [15].

Therefore, the combination of ACBP and ASLB chemotherapy might be a new anti-cancer strategy that is capable of concurrently suppressing tumor growth and improving quality of life. Our previous findings showed that an ACBP and ASLB combination treatment of MKN45 cells decreases cell proliferation and induces apoptosis (data not shown). Therefore, we speculate that ACBP is a promising candidate for GC therapy. However, the specific mechanism of tumor cell suppression remains unclear.

Long noncoding RNAs (lncRNAs) are factors in a broad range of diseases, including human cancers [16], yet their specific molecular mechanisms are poorly understood. LncRNAs are hypothesized to control cellular processes such as proliferation, development, lineage commitment, immune response, pluripotency, and differentiation [17]. Studies estimate that approximately 14,880 lncRNAs are present in humans [18]. LncRNAs have multiple functions in GC carcinogenesis [19]. Therefore, genome-wide identification of aberrantly expressed coding genes, as well as lncRNAs in the GC cells, might be crucial steps in understanding lncRNA function in GC carcinogenesis and might identify possibilities for personalized GC treatment.

We comprehensively analyzed the transcriptome of MKN45 cells treated with ACBP and ASLB, either alone or in combination, in the genome-wide scale by next-generation RNA sequencing (RNA-seq), a method that provides increased sensitivity, with the capacity to detect low-copy transcripts, novel transcripts, lncRNAs, and splice isoforms. In addition, we described changes in lncRNAs, investigated the relation between differentially expressed lncRNAs and mRNAs, and determined the biological processes and pathways that facilitate GC cell suppression by ACBP or ASLB or both to identify the regulatory molecules.

## RESULTS

### Genome-wide identification of differentially expressed LncRNA in MKN45 GC cells

**Figure 1:**
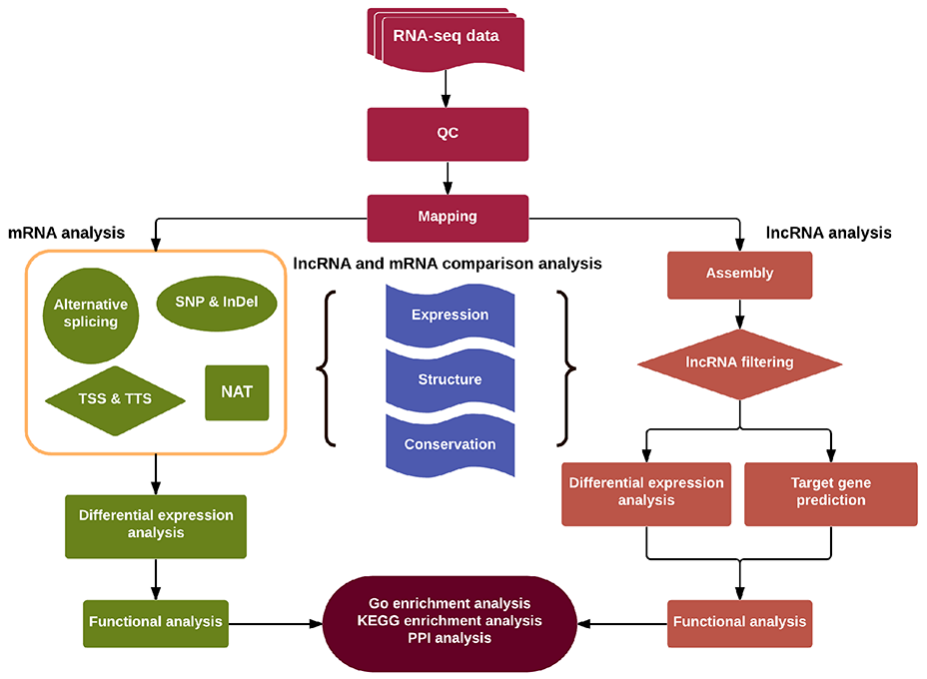
Schematic diagram of the methodology utilized for discovery and characterization of LncRNAs and mRNAs in this study

**Figure 2:**
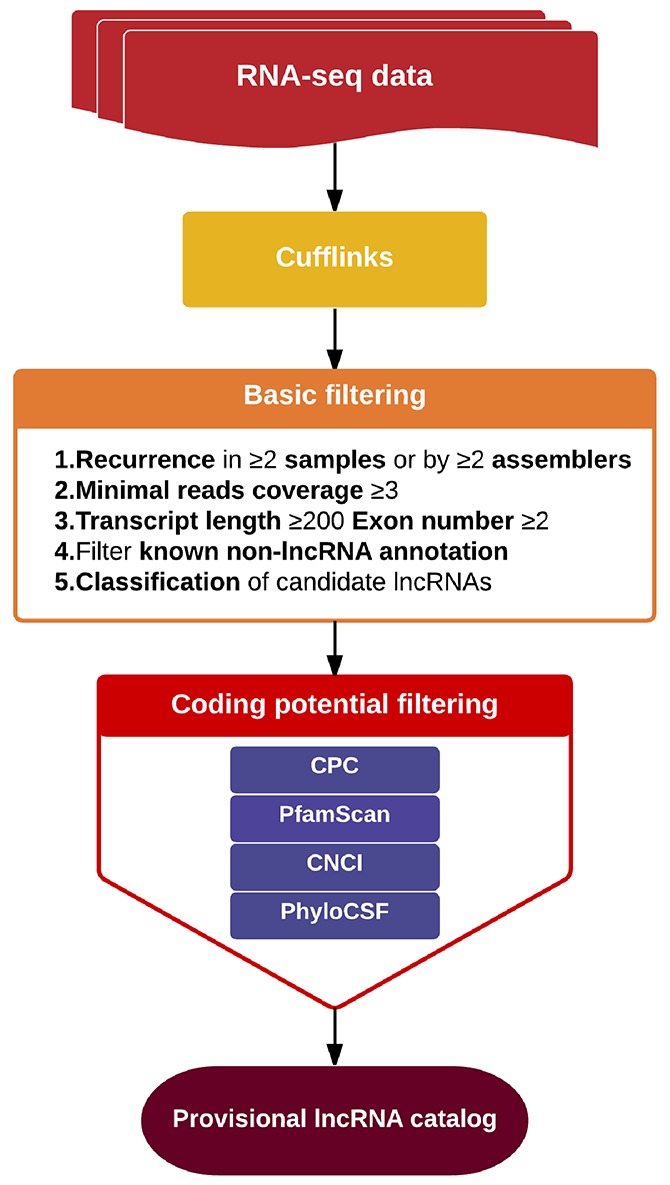
Schematic of the process of lncRNA screening

**Figure 3:**
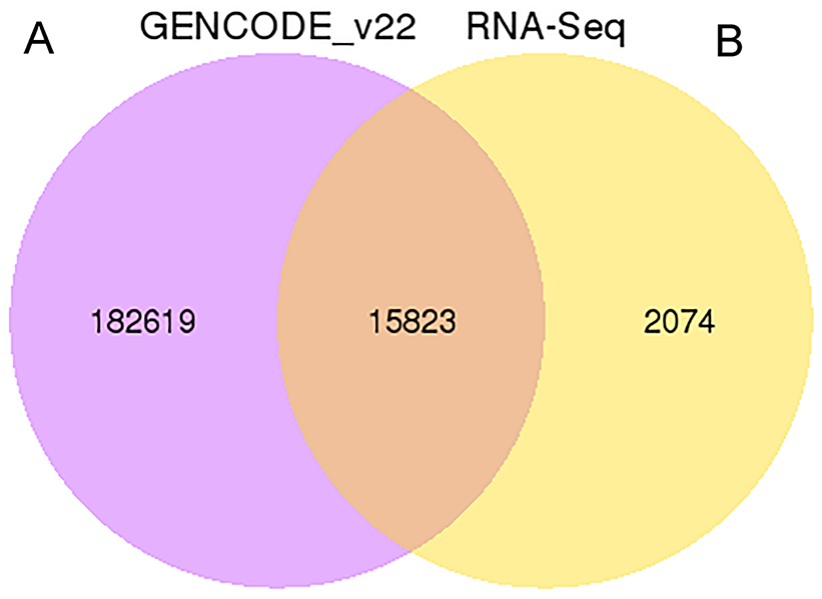
The Venn diagrams of identified lncRNA **(A)** The total number of lncRNAs identified compared with the Gencode v22 data set. **(B)** The number of lncRNAs identified by each tool.

**Figure 4:**
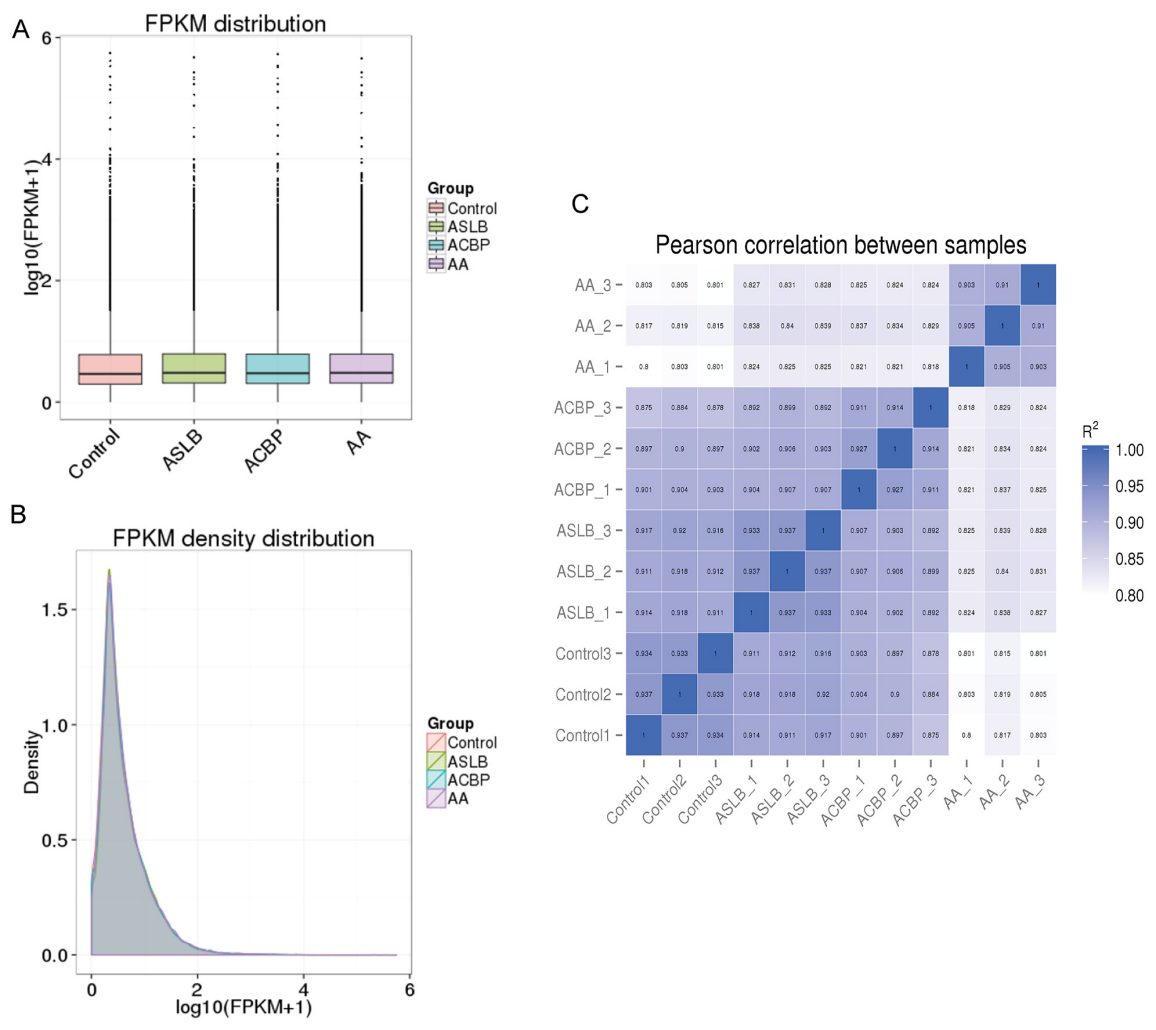
Correlation of expression patterns in each sample The box plots **(A)** and density distribution **(B)** show the expression feature of transcripts in each sample. **(C)** Calculated Pearson correlation coefficients distribution between each two samples.

We applied a computational approach and stepwise filtering procedures (Figure 1) to identify high-confidence lncRNAs from the RNA-seq cohort, with a focus on novel lncRNAs that have not been previously annotated. Twelve MKN45 GC cell samples exposed to ACBP and ASLB, alone or in combination, were analyzed [16]. Thus, 79 million to 106 million raw reads and 39 million to 53 million clean reads per sample were obtained. The clean reads were then aligned to an hg38 reference genome by TopHat software (http://ccb.jhu.edu/software/tophat/index.shtml) and assembled by Cufflinks software (http://cole-trapnell-lab.github.io/cufflinks/) to identify the known and novel transcripts on the basis of various filter criteria (Figure 2). The proportion of mapped reads identified was more than 80% of total reads sequenced, of which the uniquely mapped reads were approximately 75% to 84%. Subsequently, the mapped reads were classified by HTSeq software, and most of them were protein-coding transcripts from each sample. We used the Coding-Non-Coding-Index (CNCI), Coding Potential Calculator (CPC), Pfam-scan (PFAM), and phylogenetic codon substitution frequency (phyloCSF) tools to remove potential coding transcripts. A total of 17,897 lncRNAs were identified from RNA-seq, of which 2,074 lncRNAs were novel compared with the human genome assembly GENCODE_V22 database (Figure 3A). The number of noncoding RNAs identified by each tool is shown in Figure 3B. To identify the differentially expressed genes (DEGs) among the different exposed groups, the level of transcripts was calculated by Cuffdiff software (http://cole-trapnell-lab.github.io/cufflinks/cuffdiff/index.html). The expected number of fragments per kilobase of transcript sequence per million base pairs sequenced (FPKM) values were calculated to screen for differentially expressed RNAs. As a result, FPKM box plots and density distribution showed that the transcripts expression pattern of four kinds of exposed cells were similar (Figure 4A). We observed a correlated expression relation in each two samples with Pearson correlation coefficient > 0.8 (Figure 4B). This result suggested that the RNA-seq data from each sample were reliable and could be used for further analysis.

**Figure 5:**
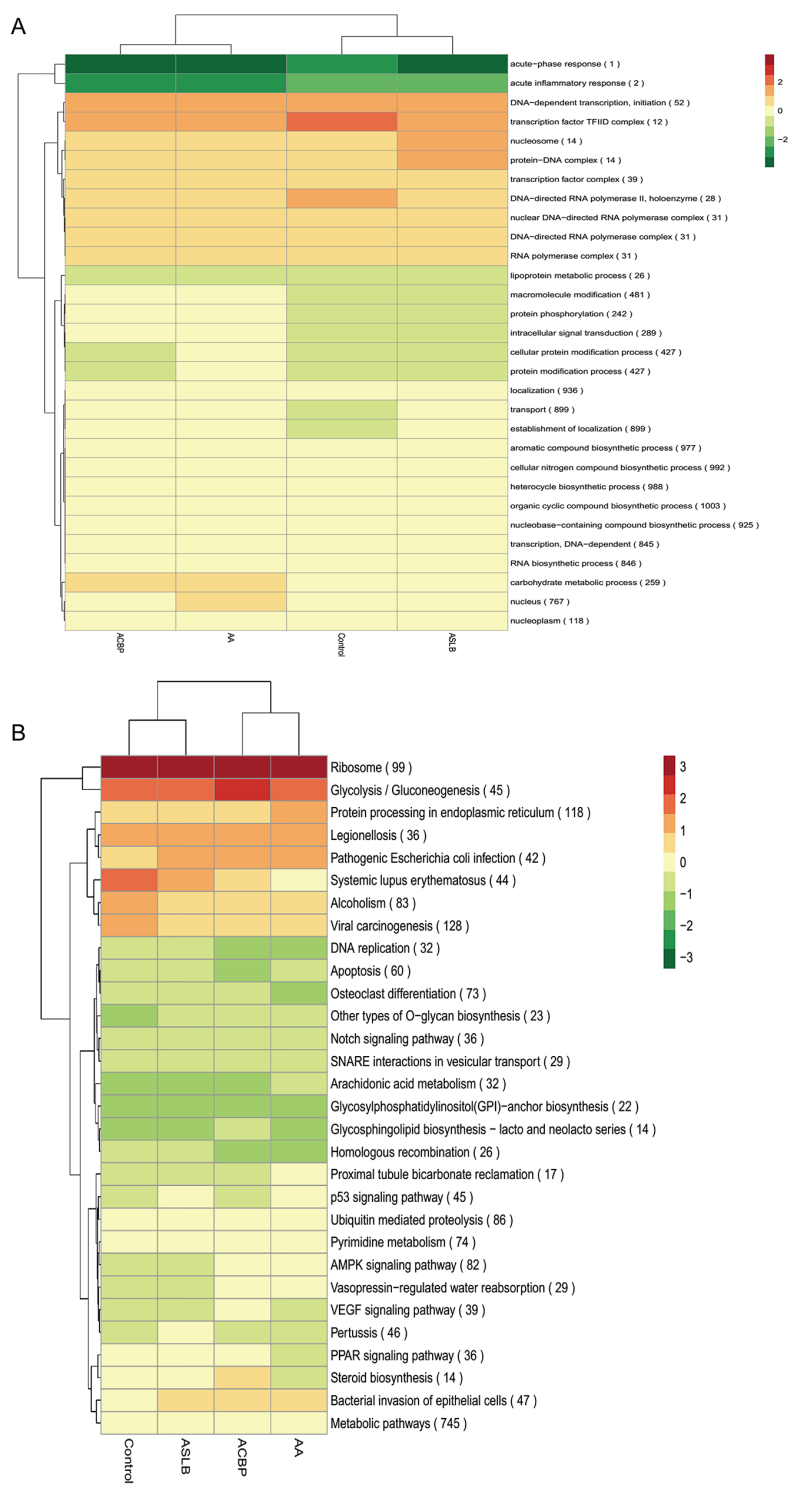
A hierarchical heat map of differentially expressed lncRNAs with transformed expression fold values by GO **(A)** and KEGG **(B)** analyses.

In general, lncRNA is a cis-acting element in mRNA expression [20]. Therefore, the genes with 100 kb up-adjacent or down-adjacent to differentially expressed lncRNAs were predicted to be their transcripts. The function of the lncRNAs was then predicted by their possible gene transcripts by application of Gene Ontology (GO) and Kyoto Encyclopedia of Genes and Genomes (KEGG) pathway analyses. The differentially expressed lncRNAs from the cells exposed to ACBP and ACPB in combination with ASLB were clustered together and were different from cells exposed to ASLB alone and from the untreated control cells (Figure 5). This result shows that the lncRNAs that promote GC cell suppression by ACBP treatment might be different from the lncRNAs that promote suppression of GC cells treated by ASLB, suggesting different mechanisms underlying GC cell suppression by ACBP and ASLB. The hierarchical analysis showed that “acute-phase response” and “acute inflammatory response” biological processes were downregulated in the GC cells treated with ACBP and ASLB, alone or in combination (Figure 5). The upregulated lncRNAs linked to suppressed GC cells with different exposure protocols were mainly correlated with transcription processes and located in the nucleosome (Figure 5). This result indicates that transcription-related lncRNAs are an important factor in the suppression of MKN45 GC cells exposed to either ACBP or ASLB. KEGG analysis indicated that metabolic pathways were utilized in the cell suppression by ACBP and ASLB.

**Figure 6:**
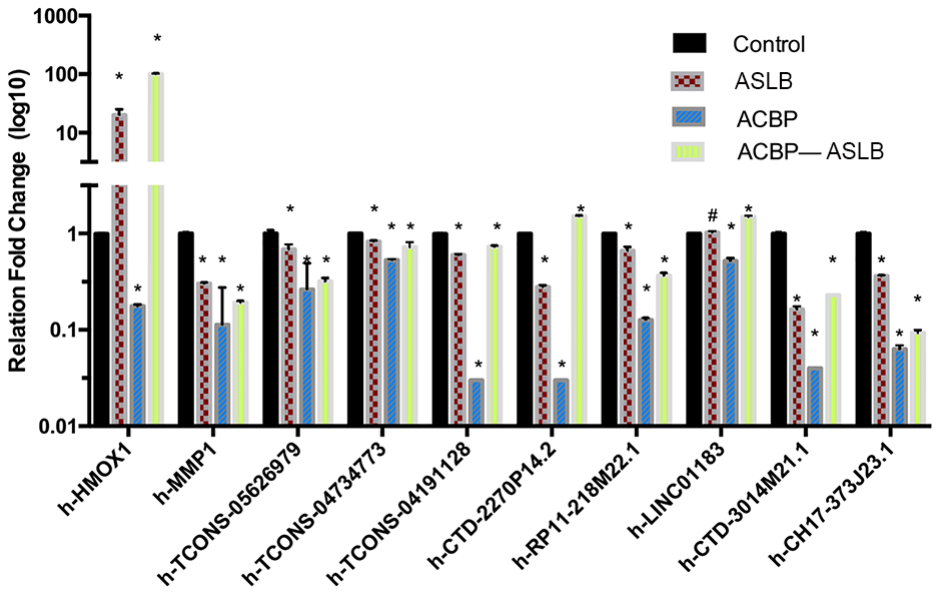
The qPCR confirmation results show consistency with the RNA-seq data Note: * indicates *P* < 0.05; ** indicates significant difference (*P* < 0.01); # indicates *P* > 0.05.

To further enhance the data reliability, we selected 10 differentially expressed transcripts (8 lncRNAs and 2 mRNAs) in which 5 were annotated by their genes (CTD-2270P14.2, RP11-218M22.1, LINC01183, CTD-3014M21.1, and CH17-373J23.1) and 3 were identified as novel for quantitative real-time PCR (RT-qPCR) analysis in MKN45 cells with different exposure protocols (Supplementary Table 1). The selected lncRNAs were differentially expressed in at least one comparison group. The RT-qPCR results showed that the expression pattern of these genes was consistent with the RNA-seq findings (Figure 6). These results suggest that the bio-computation prediction on ASLB-responsible and ACBP-responsible transcripts is reliable.

### Genomic features of lncRNA

**Figure 7:**
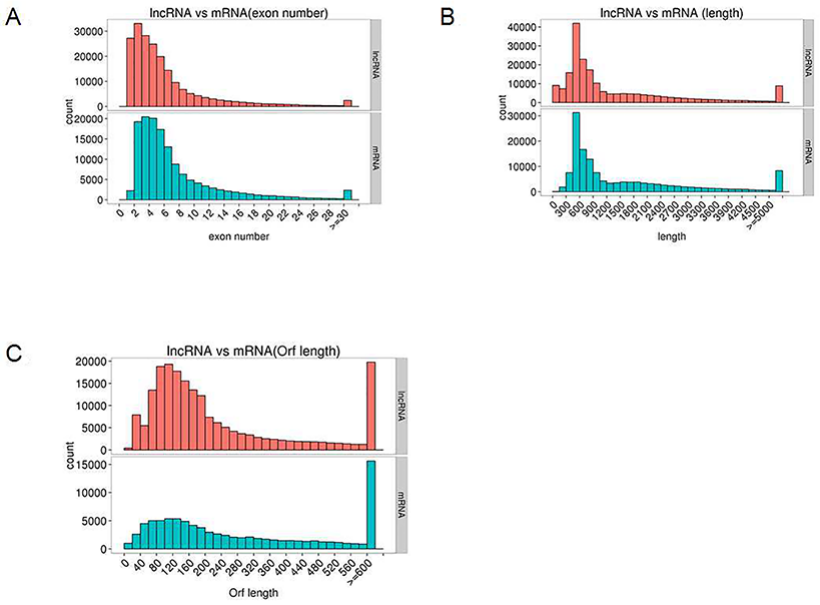
Genomic features of predicted lncRNAs and mRNAs **(A)** Exon number distribution. **(B)** Length distribution. **(C)** Orf length distribution.

**Figure 8:**
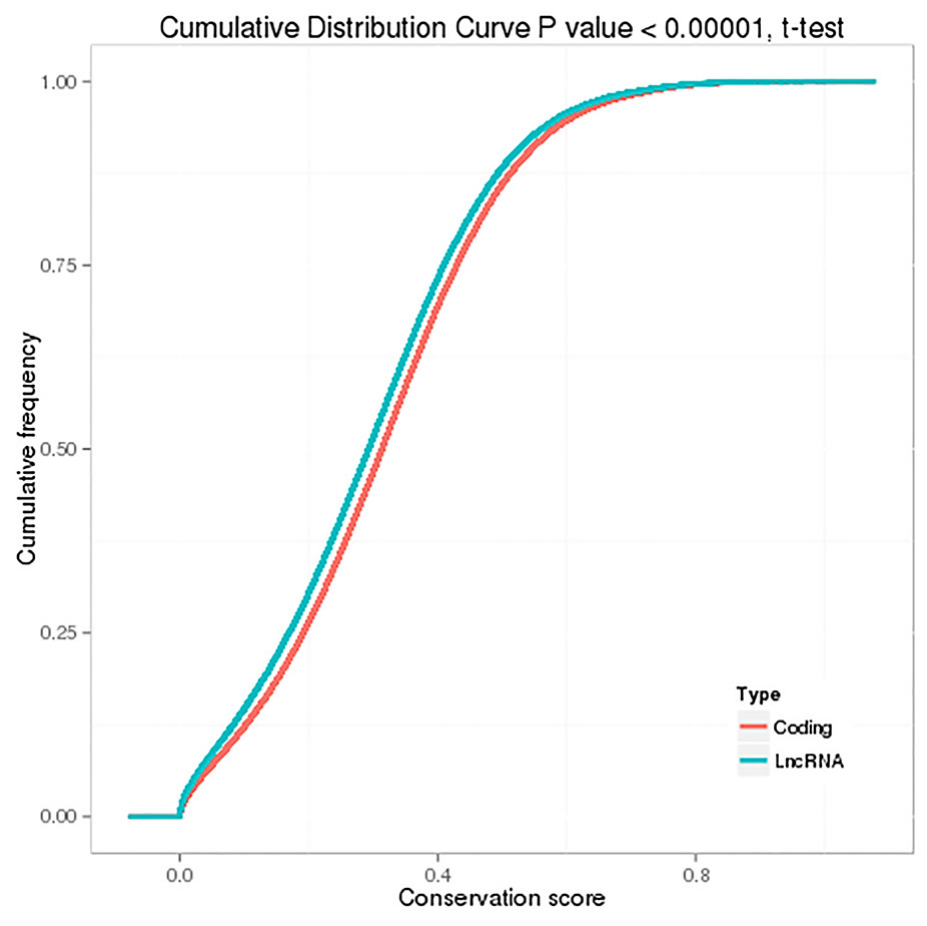
Conservation of predicted protein-coding transcripts and lncRNAs

Generally, lncRNAs are shorter in length and less conserved than protein-coding transcripts, and lncRNAs are expressed at lower levels [20]. In our study, we found that the exon number and length of identified lncRNAs were comparatively shorter than mRNA transcripts (Figure 7A and 7B). In addition, the open reading frame (ORF) length in most identified lncRNAs was shorter than 300 bp, which is consistent with lncRNA characteristics [20] (Figure 7C). We also found that the identified lncRNAs were more conserved than mRNAs (Figure 8). Furthermore, the chromosome distributions of lncRNAs identified from three differentially treated cells and untreated control cells were determined, and most reads were distributed in chromosomes 1 and 2 (Supplementary Figure 1).

### Global changes in the lncRNA response to ACBP in MKN45 cells

**Figure 9:**
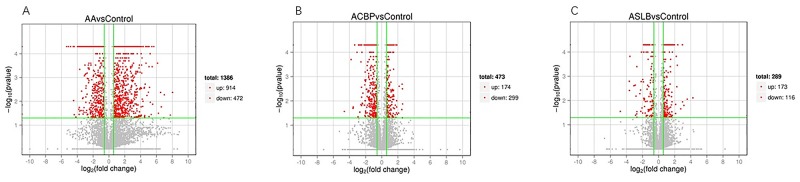
Differentially expressed lncRNA transcripts identified in ACBP **(B)**, ASLB **(C)** alone, and combined ACBP and ASLB **(A)** treated MKN45 GC cells (over 1.5-fold changes, q-value <0.05).

To elucidate global changes in transcript abundance in MKN45 GC cells in response to ACBP treatment, lncRNA expression profiling was analyzed. As shown in Figure 5, the lncRNA expression patterns between ACBP-treated cells and untreated control cells were different, according to the hierarchical clustering analysis. In total, 473 lncRNA transcripts were identified as differentially expressed (>1.5-fold change) in ACBP-treated cells when the *q*-value (adjusted *P-*value) was < 0.05 (Figure 9B). Among them, 174 and 299 transcripts were up-expressed and down-expressed in the ACBP-treated cells, respectively (Figure 9B). The 10 most upregulated and annotated lncRNA transcripts were RP11-775C24.4, RP11-65J21.3, MIR22HG, CTD-2270P14.1, MIR4697HG, SHANK3, CTC-205M6.5, RP11-50I19.2, TINCR, and RP11-29G8.3 (*q*-value < 0.05; Supplementary Table 2). The 10 most downregulated lncRNAs in ACBP treated cells were RP11-6F2.5, RP11-1008C21.2, RP11-150O12.3, RP11-553L6.5, RP11-245D16.4, H1FX-AS1, LHFPL3-AS2, RP11-61J19.5, LINC01183, and CTC-573N18.1 (*q*-value < 0.05; Supplementary Table 2). In addition, 36 novel lncRNAs were identified as differentially inhibited in the ACBP-treated cells (Supplementary Table 2).

**Figure 10:**
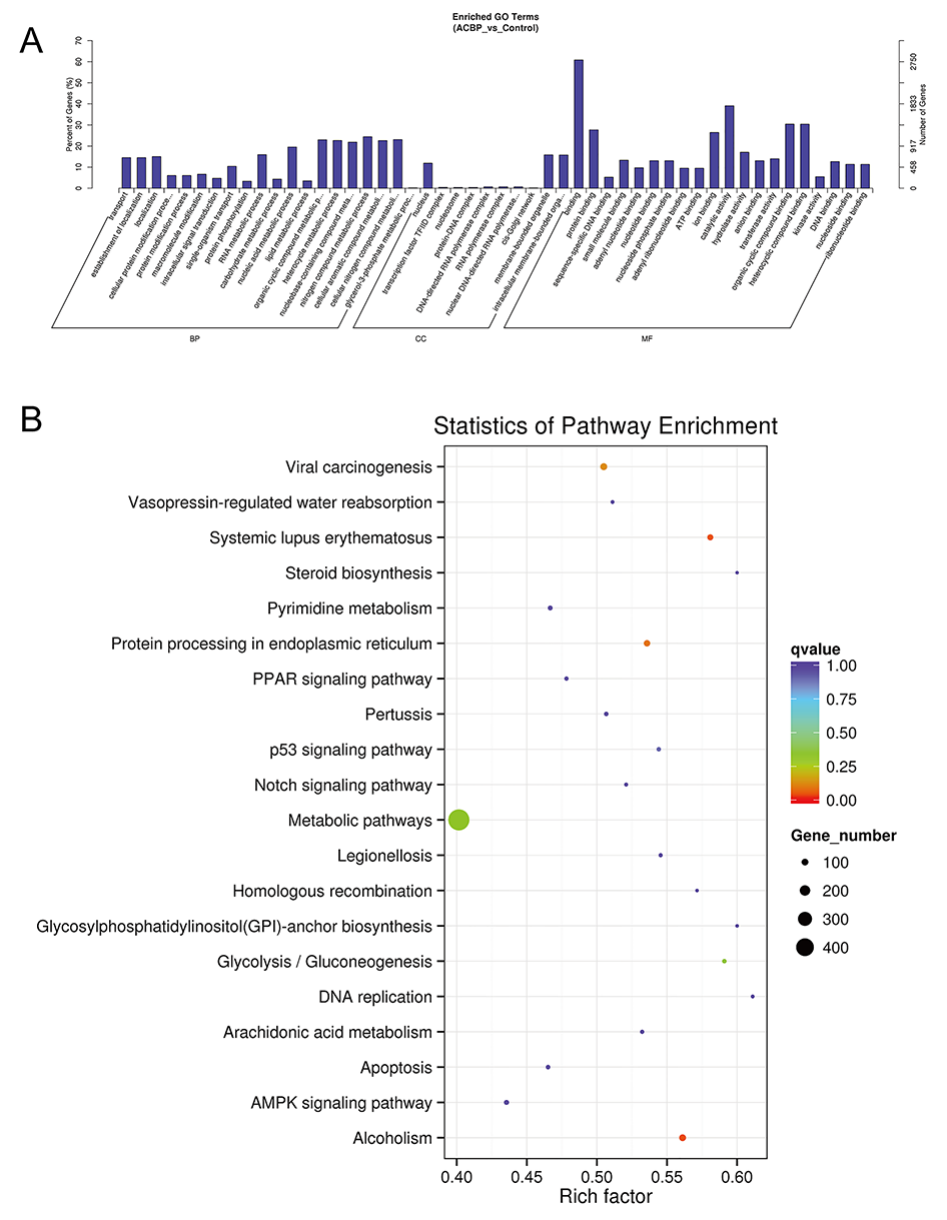
**GO (A)** and KEGG **(B)** analyses of lncRNA functions in ACBP-treated MKN45 cells.

GO analysis found that many differentially expressed genes (DEGs) were predicted to be localized in the nucleus and had protein-binding capacity, which were classified by cellular component and molecular function terms, respectively (Figure 10A). In addition, these DEGs were linked to the nitrogen compound metabolic process and the organic cellular compound metabolic process. KEGG analysis showed that viral carcinogenesis and protein processing in the endoplasmic reticulum (ER) were factors in the MKN45 cell growth suppression by ACBP (Figure 10B).

To determine the specific pathway of ACBP inhibition of MKN45 cell growth, the DEGs in the 100-kb range in the lncRNAs with >2-fold changes were analyzed by KEGG. The DEGs were all downregulated in ACBP-treated cells (*q-*value < 0.05) and linked to the cytokine-cytokine interaction pathway, the T cell receptor pathway, chemokine signaling, and the NF-kappa B, TNF, and PPAR signaling pathways. The result indicated that the suppressed expression of DEGs might be the consequence of or essential for the MKN45 cell death by ACBP treatment.

### Transcriptomic lncRNA response to ASLB

**Figure 11:**
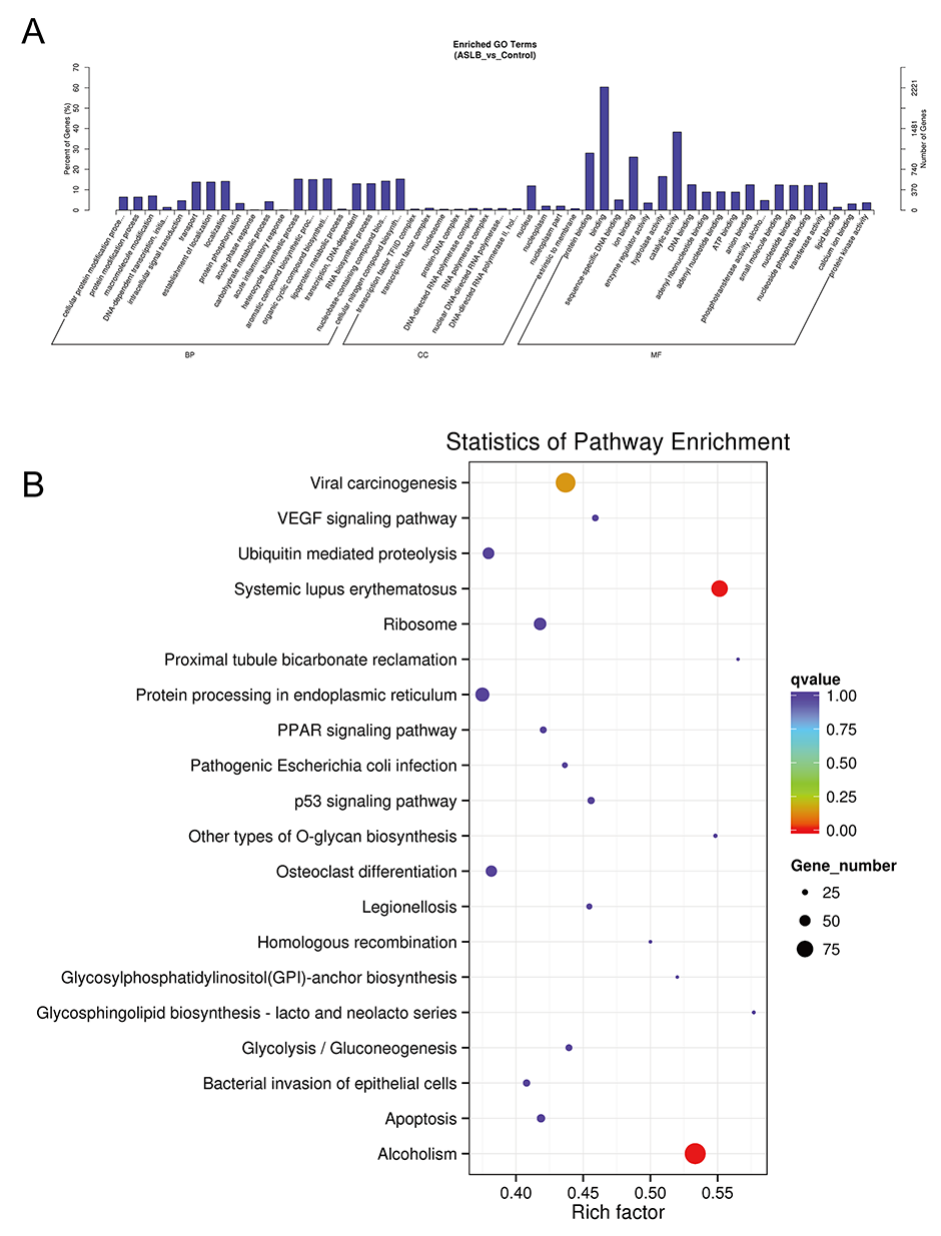
GO **(A)** and KEGG **(B)** analyses of lncRNA functions in ASLB-treated MKN45 cells.

To understand the mechanism of MKN45 cell death by treatment with the chemotherapy drug ASLB, the lncRNA transcript expressions were analyzed by RNA-seq. A total of 289 differentially expressed lncRNAs were detected in ASLB-treated cells, including 173 upregulated and 116 downregulated lncRNAs (*q*-value < 0.05, >1.5-fold change) (Supplementary Table 2). The number of identified lncRNAs was fewer than the number identified in other exposure protocols. Most of the differentially expressed lncRNAs were also found to localize in the nucleus and participate in the DNA-dependent transcription and transport processes and function in the catalytic activity, protein binding, and other important signaling pathways by GO prediction (Figure 11A). KEGG analyses revealed viral carcinogenesis in ASLB-induced MKN45 cell death (Figure 11B). Compared with the lncRNAs identified from ACBP-treated cells, the number of differentially expressed lncRNAs was lower in ASLB-treated cells. This result indicates that fewer transcripts are eliminated by ASLB-induced GC cell death, and the mechanism for cell suppression might be different for ACBP and ASLB treatments.

### Transcriptomic lncRNA response to the combination of ASLB and ACBP in MKN45 cells

**Figure 12:**
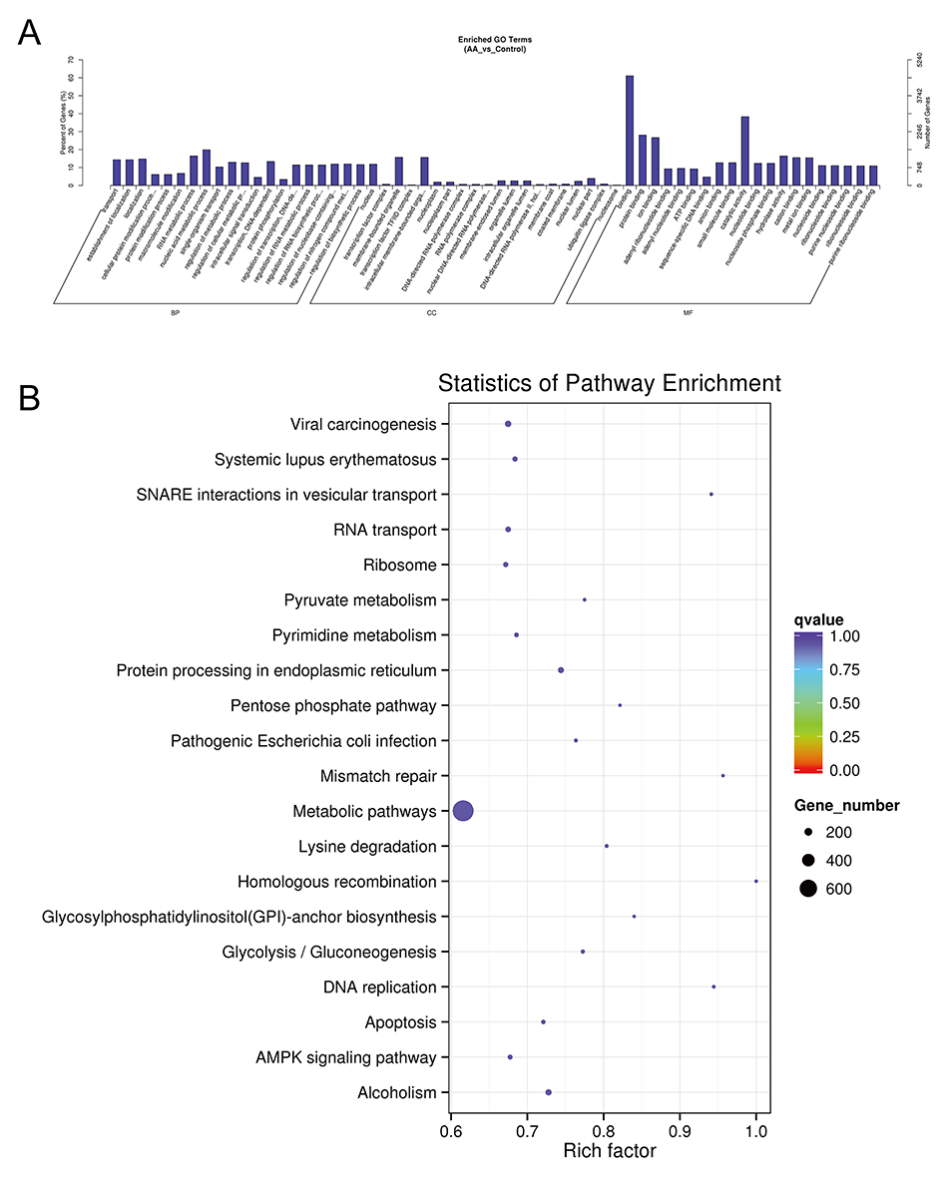
GO **(A)** and KEGG **(B)** analyses of lncRNA functions in combined ACBP and ASLB treated MKN45 cells.

Because ACBP and ASLB combination treatment in MKN45 GC cells decreases cell proliferation and induces apoptosis, we elucidated the mechanism and analyzed the transcriptomic changes in the MKN45 cells in response to the combination of ASLB and ACBP. As shown in Figure 9A, 1,386 lncRNAs were identified in the cells exposed to combination treatment, including 914 upregulated and 472 downregulated lncRNAs (>1.5-fold, *q* < 0.05). We observed that the number of differentially expressed lncRNAs identified from the combination treatment was higher than the number identified from ACBP and ASLB monotherapy, indicating that combination treatment disrupts more lncRNAs in GC cell death. Pathway analyses by GO and KEGG were used to reveal the pathways within differentially expressed transcripts. Differentially expressed lncRNA transcripts were mainly localized in the nucleus and in membrane-bound organelles, and were enriched in biological processes such as metabolic pathways, molecular function of protein, ion binding, and catalytic enzyme activity (Figure 12).

### Carcinogenesis-related lncRNAs with aberrant expression in differentially exposed cells

Studying the aberrantly expressed lncRNAs that activate signaling pathways might deepen our understanding of the occurrence and development of GC and provide new insights for further therapy. We analyzed the aberrantly expressed lncRNAs from different exposed MKN45 cells. The annotated lncRNA HOX transcript antisense RNA (HOTAIR), previously reported to be highly expressed in many cancers, including GC [21], was downregulated in MKN45 cells treated with combined ACBP and ASLB compared with untreated control cells (4.3-fold changes, *P* < 0.05). HOTAIR expression decreased 5.6-fold in the ACBP and ASLB combination-treated cells compared with HOTAIR expression in ASLB-treated cells (Supplementary Table 3). However, HOTAIR expression was not significantly different in ACBP-treated or ASLB-treated cells when compared with other exposure protocols. This result suggests that suppression of HOTAIR by ACBP and ASLB combination treatment in MKN45 cells might inhibit tumor cell growth in a synergistic manner, or, potentially, HOTAIR downregulation is the consequence of transcriptomic expression change induced by the inhibition of MKN45 cell growth. These data also indicate that HOTAIR might be a factor in GC cell death induced by combined ACBP and ASLB exposure, but might not be a factor in ACBP-induced GC cell death. In addition, the expression of metastasis-associated lung adenocarcinoma transcript 1 (MALAT-1) lncRNA, which is significantly over-expressed in various cancers [22], was found to be upregulated with 1.58-fold change (Supplementary Table 3, *P* < 0.05) in combined ACBP and ASLB treated cells compared with untreated control cells, and MALAT-1 was not detected in other cells by different exposure protocols. Similarly, lncRNA H19, which has been reported to be upregulated in diverse human cancers [23], was highly expressed in ACBP and ASLB treated cells (2.04-fold change, *P* < 0.05, Supplementary Table 3). Long intergenic noncoding RNA 152 (LINC00152) was found to be downregulated in ACBP-treated, ASLB-treated, and combination-treated cells (Supplementary Table 3). The expression of the above lncRNAs reported in GC development showed different patterns, suggesting that HOTAIR and LINC00152 expressions are suppressed and both might promote MKN45 cell death in ACBP and ASLB combination therapy.

Furthermore, the gastric carcinoma high expressed transcript 1 (GHET1) was downregulated in combined ACBP and ASLB treated cells compared with untreated control cells and ASLB-treated cells (4.9-fold and 3.6-fold, respectively, *q* < 0.05, Supplementary Table 3). However, GHET1 was not detected with altered expression in ACBP-treated or ASLB-treated cells. The downregulation of GHET1 in combination-treated GC cells suggested that ACBP and ASLB synergistically suppressed GHET1 expression and subsequently acted in tumor cell proliferation. In addition, the expression level of the MIR210 host gene (MIR210HG), which is considered a diagnostic biomarker for glioma [24] was significantly downregulated in combined ACBP and ASLB treated cells when compared with other singly treated cells and untreated control cells (Supplementary Table 3). The miR-17-92a-1 cluster host gene (MIR17HG), which is downregulated in GC [25], was found to be highly expressed in combined ACBP and ASLB treated cells when compared with the other treatment groups (Supplementary Table 3). MIR22HG, which is downregulated in lung carcinoma [26], was found to be upregulated in ACBP monotherapy cells and ACBP and ASLB combination-treatment cells (Supplementary Table 3). These results indicate that MIR17HG and MIR22HG might promote ACBP-induced GC cell death and can be selected for directed GC therapy.

We searched the other known lncRNAs in our data set and found several small nucleolar RNA host gene family members with differential expressions. The small nucleolar RNA host genes promote nucleosome remodeling and histone deacetylation complex formation. The expression of SNHG5 was downregulated in GC [27] and linked with tumor cell proliferation and metastasis [27]. We found that SNHG5 was upregulated in ACBP-treated cells and ASLB-treated cells (Supplementary Table 3). Besides SNHG5, the expression of SNHG1 and SNHG12 was also increased in combination-treated cells, especially SNHG12 (Supplementary Table 3).

In addition, we found that lung cancer associated transcript 1 (LUCAT1), tissue differentiation-inducing non-protein coding RNA (TINCR), and taurine upregulated gene 1 (TUG1) were upregulated in combined ACBP and ASLB treated cells (Supplementary Table 3). LUCAT1 was enhanced in cisplatin-resistant A2780-DR high-grade serous ovarian cancer cells [28]. TINCR was upregulated in human gastric carcinoma [29], and TUG1 was upregulated in many cancer tissues and cells [30]; therefore, the increased expression of LUCAT1, TINCR, and TUG1 might not be specific to ACBP and ASLB treatments.

## DISCUSSION

In the clinic, most patients with advanced and metastatic GC are treated with ASLB chemotherapy [31]. However, ASLB-induced peripheral neuropathy and cell toxicity are major concerns [32]. Identification of agents with potent antitumor activity and low cell toxicity would be beneficial to patients. Previously, we found that ACBP exhibited high efficacy and low toxicity in the inhibition of MKN45 cell growth. However, the specific mechanism of cancer cell growth inhibition by ACBP has not been elucidated. A number of studies have demonstrated dysregulated levels of lncRNAs in various types of cancers, including GC [31]. Furthermore, lncRNAs can induce gene expression at epigenetic, transcription, and post-transcription levels [33], thus lncRNAs have potential as diagnostic markers and selection for directed therapy in cancers.

Advances in high-throughput biotechnologies have led to the exponential growth of transcriptomic profiles. However, because cancer genomes are highly unstable, many cancer-associated alterations are not the cause, but the consequence, of tumorigenesis. Analyses of genome-wide profiles identified by RNA-seq using various bioinformatics approaches can reveal specific signaling pathways in human GC treated by effective drugs. Hence, to identify the key regulatory molecules and the underlying mechanism in the suppression of MKN45 GC cells by ACBP, we generated global lncRNA and mRNA expression profiles from ACBP-treated MKN45 GC cells using the RNA sequencing method.

ASLB has been widely used in the chemotherapeutic regimes for many types of cancer, including GC [34]. Despite several biomarkers suggested to be factors in the response of cancer cells to ASLB therapy, the underlying mechanism of ASLB-induced cancer cell death remains unknown. To identify regulatory lncRNAs and their functions in the GC cell response to ASLB, the genome-wide expression of transcripts was investigated in ASLB-treated MKN45 cells by use of RNA-seq. This report is the first to systematically analyze lncRNA expression in ACBP-treated and ASLB-treated MKN45 cancer cells.

We identified 1,386 differentially expressed lncRNAs (>1.5-fold) in combined ACBP and ASLB treated cells compared with untreated control cells, 1,254 differentially expressed lncRNAs compared with ASLB-treated cells, and 1,214 differentially expressed lncRNAs compared with ACBP-treated cells. The RT-qPCR experiment showed consistent results with the RNA-seq data. This outcome indicates that data from our RNA-seq analysis are reliable for further analysis. From the results, we identified that the number of differentially expressed lncRNA transcripts from combined ACBP and ASLB treatment was higher than the numbers from either single treatment. In addition, most of differentially expressed lncRNAs were not functionally characterized. These differentially expressed transcripts identified might be factors in the response to ACBP and ASLB treatments. The result indicates that ACBP and ASLB might synergistically alter lncRNA and mRNA transcript expressions. These transcripts might be new biomarkers for evaluation of the effects of ACBP and ASLB treatment in GC in future clinical trials and might be selected for directed therapy. This report is also the first to reveal the molecular mechanism of ACBP and ASLB in GC.

Evidence suggests that lncRNAs promote expression at the epigenetic, transcriptional, or post-transcriptional level in many cancers. The identification of lncRNAs has expanded our notions of the transcriptome complexity and gene regulatory network in ACBP-treated or ASLB-treated GC cells. Therefore, we performed the GO and KEGG pathway analyses to identify the possible regulatory molecules and signaling pathways in the response of MKN45 cells to ACBP and ASLB treatment. This knowledge might provide new insights into the occurrence and development of GC, and suggest potential therapeutic strategies. The KEGG hierarchical clustering indicated that different lncRNAs were altered at the transcription level in MKN45 cells treated with ACBP or ASLB. The regulatory mechanism of the ACBP therapy effect on GC cells might be different from the mechanism of ASLB. GO classification analyses showed that differentially expressed lncRNAs were localized in the nucleus in three exposure protocols. In addition, cellular localization of lncRNAs was membrane-bound, and intracellular membrane-bound organelles occurred in protocols that included ACBP-treated cells but not in protocols that included ASLB-treated cells. This result indicates that altered lncRNAs expressed in ACBP-treated MKN45 cells can function in the nucleus as well as in membrane-bound organelles. LncRNAs are less likely to be oncogenes because of the lack of RNA expression or genetic alterations in cancer; therefore, the predicted genes were used for lncRNA function analysis. Consequently, the p53 signaling pathway was activated in the ACBP-treated GC cells, as determined by KEGG analysis, which indicated that ACBP inhibited GC cell proliferation through p53 signaling. In addition, DNA replication and cell cycle processes were also enriched in ACBP-treated cells. This result is consistent with our previous report that showed cell cycle arrest in ACBP-treated cancer cells. The altered expressed lncRNA in ACBP-treated cells promote the gene expression that activates the cell cycle, DNA replication processes, and the p53 cell signaling pathway, to suppress GC cell growth.

We investigated the known lncRNAs expression in differentially treated cells. Notably, several known lncRNAs expressions were identified as downregulated in combined ACBP and ASLB treated cells compared with untreated control cells or ACBP-treated cells. We demonstrated that the downregulation of HOTAIR might be a factor in GC cell death induced by the combination of ACBP and ASLB. HOTAIR is an oncogenic factor and has been used as a prognostic biomarker in different cancer types [35]. HOTAIR overexpression correlates with many cancers and it is linked to cancer cell resistance to cisplatin. Liu et al. [36] found that knockdown of HOTAIR re-sensitizes the responses of A549/DDP cells to cisplatin. HOTAIR can function as competitive endogenous RNAs (ceRNAs) in gastric cells by recruiting the microRNAmiR-331-3p, disrupting HER-2 expression [37]. In addition, we found MIR210HG, LINC00152, and GHET1 were downregulated in combination ACBP and ASLB treated cells when compared with control cells (−41.8, −3.87, and −19.9 fold, *P* < 0.05). As previously reported, miR210HG levels were higher in glioma patients and predicted complementary binding with BMP1 [24]. LINC00152 was upregulated in GC and promoted GC cell cycle progression by suppressing expression of p15 and p21 via binding to enhancer of zeste homolog 2 (EZH2) [38]. In addition, Linc00152 activates PI3K/Akt pathway by directly binding to EGFR [39]. GHET1 was overexpressed in GC tissues, and functional analysis studies found GHET1 promoted GC cell proliferation by increasing the oncogene c-myc mRNA stability and expression via interaction with IGF2 mRNA-binding protein 1 (IGF2BP1) and c-myc mRNA [40]. ACBP and ASLB induced MKN45 cell death resulted in part in the downregulation of the lncRNAs HOTAIR, MIR210HG, LINC00152, and GHET1. Hence, these data indicate that these lncRNAs might be important in the carcinogenesis and progression of GC and can be selected for directed therapy.

ASLB induces DNA damage through cross-linking, and lncRNAs are factors in chemical-induced DNA-damage response, which suggests that lncRNAs might also enhance ASLB resistance in GC chemotherapy. We found that the regulatory mechanism of the ASLB effect in MKN45 cell death might be different from the ACBP effect. Besides the several known lncRNAs that were downregulated in ACBP-treated MKN45 cells, many novel lncRNAs were also identified, which indicate that ACBP-induced suppression of MKN45 cell proliferation is likely promoted by a variety of lncRNAs at the transcriptional level. The specific function of these novel lncRNAs must be investigated further. Our work provides potential biomarkers for ACBP sensitivity in the MKN45 GC cell line and a focal point for combination therapy with ASLB.

We have identified a substantial number of ACBP-specific and ASLB-specific lncRNAs by the genome-wide scale RNA-seq method, and imputed their potential functions in ACBP-induced and ASLB-induced MKN45 cell death. Moreover, our results provide potential clues to identify the mechanisms of biologically active peptides in gene suppression and cancer therapy. Because the functions of lncRNAs in carcinogenesis and chemotherapy are not fully understood, this analysis provides information for future studies.

## MATERIALS AND METHODS

### Cell culture

The human GC cell line MKN45 was purchased from the Cell Resource Center, Institute of Basic Medical Sciences, Chinese Academy of Medical Sciences, Peking Union Medical College (Beijing, China) and maintained in the Clinical Medical Center of Affiliated Hospital (Inner Mongolia Medical University, Huhhot, China). The cells were cultured in RPMI 1640 Medium (Invitrogen, Carlsbad, CA, USA) supplemented with 10% fetal bovine serum (FBS; HyClone, Melbourne, Victoria, Australia) and 1% penicillin–streptomycin (Invitrogen, Carlsbad, CA, USA). All cells were maintained in a humidified 5% CO_2_ incubator at 37° C. MKN45 is a poorly differentiated human gastric adenocarcinoma cell line exhibiting more than 90% cancer stem-like properties.

### Production and purification of ACBPs

ACBPs were prepared and purified as previously reported [41]. A concentration of 20 μg/mL was adapted for the treatment of cells throughout the study.

### Cells exposure protocols

ASLB was purchased from Jiangsu Aosaikang Pharmaceutical Co., Ltd. (Jiangsu Province, China) and was dissolved in dimethyl sulfoxide (DMSO) as a stock solution. Experiments were conducted with human GC MKN-45 cells. Plating density was 1 × 10^6^ cells/mL. After seeding for 24 hours, the cells were exposed to 20 μg/mL induced ACBP (Group A), 15 μg/mL ASLB (Group B) and 10 μg/mL induced ACBP combined with 7.5 μg/mL ASLB (Group C). The negative control group was treated with normal saline. The cells were then collected after exposure for 36 hours. There were three biological replicates for each type of exposure.

### RNA isolation and sequencing

The collected cells were used for transcriptomic analysis. There were three biological replicates for each treatment. RNA isolation and sequencing were performed by Novogene Bioinformatics Technology Co., Ltd (Beijing, China). Isolation of total RNA was performed by use of TRIzol Reagent following the manufacturer’s instructions, and then genomic DNA was removed by use of DNase I (Invitrogen, Thermo Fisher Scientific, Carlsbad, CA, USA). RNA quality was determined by an Agilent Bioanalyzer 2100 (Agilent Technologies, Inc., Santa Clara, CA, USA), and concentration was measured by the ND-2000 Spectrophotometer (NanoDrop Technologies, Wilmington, DE, USA). The mRNA was isolated by use of oligo-dT beads, and then fragmented in a fragmentation buffer. The short mRNA fragments were used as templates to synthesize first-strand cDNA with random hexamer primers, and then the second-strand cDNA was synthesized by use of dNTPs, DNA polymerase I, and response buffer. The double-stranded cDNAs were purified by use of AMPure XP beads, and then used for end reparation and “A” base addition and finally were ligated with sequencing adapters. The adaptor-ligated fragments were size selected by use of AMPure XP beads. After quantification with a Qubit 2.0 Fluorometer (Life Technologies, Carlsbad, CA), cDNAs were used for PCR amplification and sequenced as 2 × 120 bp paired-end reads on an Illumina HiSeq 2000 Sequencer (Illumina, San Diego, CA, USA).

### Sequence tag preprocessing and mapping

Sequence tag preprocessing was performed according to a previously described protocol with some modifications [42]. Raw reads were cleaned by removing reads with adaptors, low-quality (>50%), or a high proportion unknown bases (>10%) in a read. Clean data were mapped to the hg38 reference genome from the Genome Browser Gateway (http://genome-asia.ucsc.edu/cgi-bin/hgGateway?hgsid=471475425_tssn1UOOTZ1feg0VNawOcoLJsnFk) by Bowtie 2 software (http://bowtie-bio.sourceforge.net/bowtie2/index.shtml), with a maximum allowance of two nucleotide mismatches.

### Gene expression calculation and pathway analysis

Gene expression was calculated according to the RPKM (reads per kilobase transcriptome per million mapped reads) method by HTSeq software [22] (http://www-huber.embl.de/HTSeq). Differentially expressed genes were selected based on *P*-value < 0.05. KEGG pathway analysis was performed by KOBAS 2.0 (http://kobas.cbi.pku.edu.cn/) and the false discovery rate (FDR) corrected *P*-value (*q*-value) cut-off was set at 0.05.

### Quantitative real-time polymerase chain reaction

To confirm the findings of the RNA-Seq assay, a separate exposure experiment was conducted with the same exposure protocol as described above. MKN45 cells were exposed to ACBP and ASLB, alone or in combination, for 36 hours, and then cells were collected for RT-qPCR. Isolation of total RNA, first-strand cDNA syntheses, and RT-qPCR were performed by use of commercial kits according to the instructions of the respective manufacturers. The isolation of total RNA was performed by use of TRIzol Reagent (Takara Bio Inc., Shiga, Japan) following the manufacturer’s instructions. RNA concentration and quality were assessed by use of a NanoDrop 2000 UV-Vis Spectrophotometer (Thermo Fisher Scientific, Carlsbad, CA, USA). A 1-μg quantity of total RNA was used for reverse transcription with a PrimeScript RT Reagent Kit (Thermo Fisher Scientific, Carlsbad, CA, USA). RT-qPCR was performed by use of Fast SYBR Green Master Mix (Thermo Fisher Scientific, Carlsbad, CA, USA), following the manufacturer’s instructions, and a melting curve was utilized to determine purity and specificity of PCR productions in each assay. Sequences of primers were designed and synthesized by Sangon Biotech (Shanghai) Co. Ltd, China (Supplementary Table 1). The expression of glyceraldehyde 3-phosphate dehydrogenase (GAPDH) and h-RPS20 were used as the internal controls for mRNAs and lncRNAs, respectively, to normalize the qPCR results to minimize variation between and among analyses. Thermal cycling was set at 94° C for 2 minutes, followed by 40 cycles of 94° C for 20 seconds and 60° C for 34 seconds. RT-qPCR data were presented as fold change (log2) relative to control. There were three biological replicates for each concentration.

### Statistical analysis

All data for gene expression (RT-qPCR) were analyzed by Statistical Program for Social Science (SPSS) 18.0 software (SPSS, Chicago, IL) and GraphPad Prism 6.0 (GraphPad Software, La Jolla, CA). One-way analysis of variance was used to determine significant differences between the control group and exposure group. *P* value < 0.05 was considered statistically significant.

## SUPPLEMENTARY MATERIALS FIGURE AND TABLES






