# Supplementary material for: Selecting lncRNAs in gastric cancer cells for directed therapy with bioactive peptides and chemotherapy drugs
Source: Oncotarget. 2017 Sep 18;8(49):86082–97. doi: 10.18632/oncotarget.20977 (PMC5689669; doi:10.18632/oncotarget.20977)
Supplement: Supplementary file 1 [file oncotarget-08-86082-s001.pdf]

Selecting lncRNAs in gastric cancer cells for directed therapy with bioactive peptides and chemotherapy drugs

SUPPLEMENTARY MATERIALS

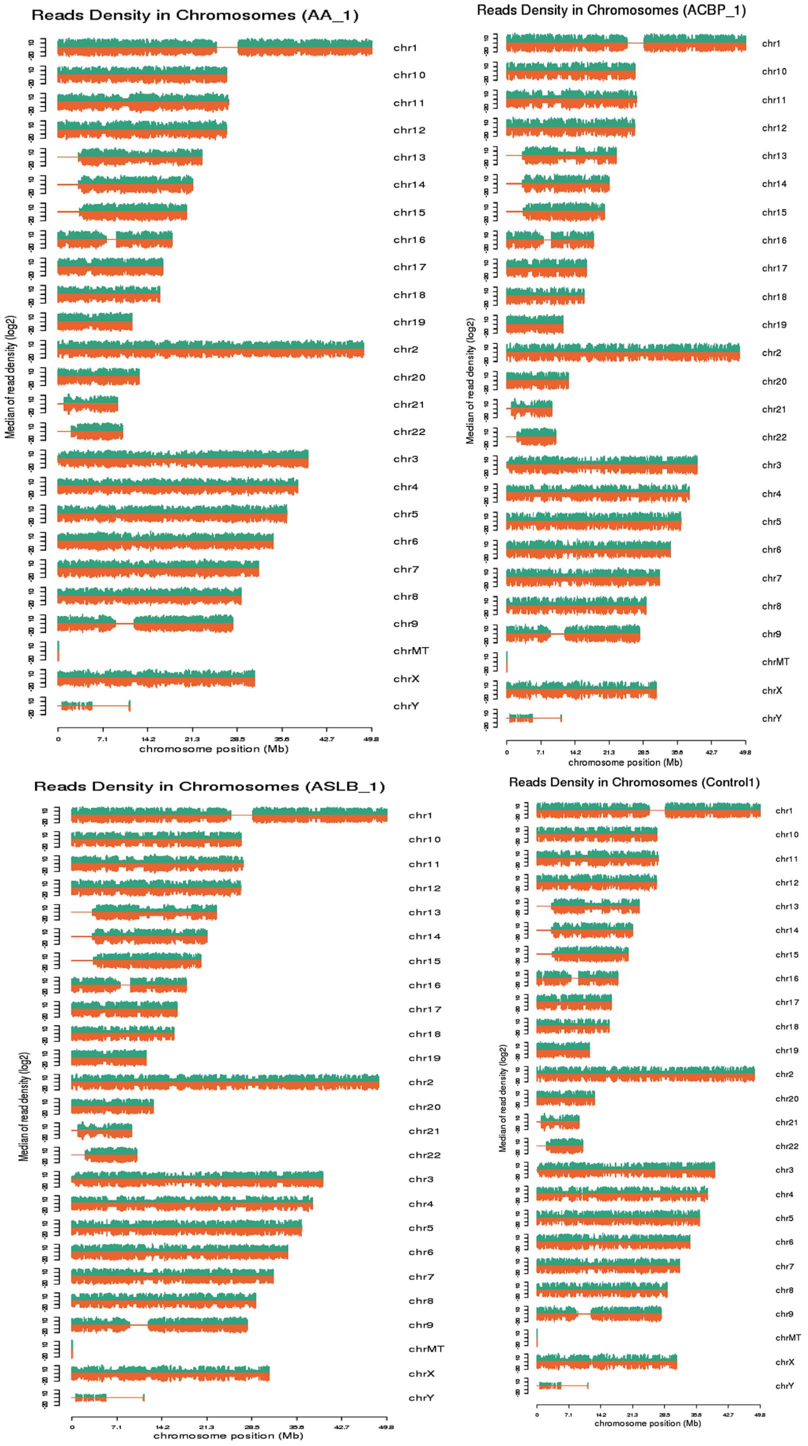

Supplementary Figure 1: Read density in chromosomes.

Supplementary Table 1 : The list of selected lnRNAs and mRNAs for validation by qPCR

Number	Transcript ID	Transcript type	Gene ID	Gene name	Length
1	ENST00000623937.1	Annotated_lncRNA	ENSG00000279901.1	CTD-2270P14.2	1659
2	ENST00000318291.4	Annotated_lncRNA	ENSG00000177406.4	RP11-218M22.1	2194
3	ENST00000453666.2	Annotated_lncRNA	ENSG00000225329.3	LHFPL3-AS2	923
4	ENST00000513708.1	Annotated_lncRNA	ENSG00000230561.3	LINC01183	663
5	ENST00000570269.1	Annotated_lncRNA	ENSG00000259976.1	RP11-553L6.5	1679
6	ENST00000624705.1	Annotated_lncRNA	ENSG00000279602.1	CTD-3014M21.1	1321
7	ENST00000618589.1	Annotated_lncRNA	ENSG00000276216.1	CH17-373J23.1	347
8	TCONS_05626979	Novel_lncRNA	XLOC_1828515	—	1825
9	TCONS_04734773	Novel_lncRNA	XLOC_1552256	—	716
10	TCONS_04191128	Novel_lncRNA	XLOC_1370458	—	8470
11	ENST00000216117	mRNA	ENSG00000100292	HMOX1	1822
12	ENST00000315274	mRNA	ENSG00000196611	MMP1	1970

Supplementary Table 2: The list of differentially expressed lncRNAs between ACBP, ASLB, and combined ACBP and ASLB treatment cells and control cells

See Supplementary File 1

Supplementary Table 3: The altered expression (log2 value) of known lncRNAs between each two comparisons of different exposed cells

See Supplementary File 2
