# Supplementary material for: Selecting lncRNAs in gastric cancer cells for directed therapy with bioactive peptides and chemotherapy drugs
Source: Oncotarget. 2017 Sep 18;8(49):86082–97. doi: 10.18632/oncotarget.20977 (PMC5689669; doi:10.18632/oncotarget.20977)
Supplement: Supplementary file 3 [file oncotarget-08-86082-s003.docx]

**Supplementary Table 3: The altered expression (log2 value) of known lncRNAs between each two comparisons of different exposed cells**

| **Known lncRNAs** | **AA/CON** | **ACBP/CON** | **ASLB/CON** | **AA/ACBP** | **AA/ASLB** | **ASLB/ACBP** | **Function** | **Related cancer types** |
| --- | --- | --- | --- | --- | --- | --- | --- | --- |
| **GHET1** | −2.3 | −0.86 | / | / | −1.86 | / | Proliferation, invasion, cell cycle, tumorigenicity | Gastric, bladder, and colorectal cancer |
| **LUCAT1(SCAL1)** | 2.47 | 1.33 | 1.58 | 1.14 | 0.89 | / | Proliferation | Ovarian and lung cancer |
| **TINCR** | 2.39 | 1.46 | / | 0.93 | 2.51 | −1.58 | Proliferation, apoptosis, tumorigenicity, cell cycle | Gastric, bladder, and colorectal cancer, squamous cell carcinoma |
| **TUG1** | 2.83 | / | 0.46 | 0.66 | 0.45 | / | Proliferation, migration, invasion, apoptosis, tumorigenicity, metastasis | Prostate, ovarian, colon, lung and bladder cancer, osteosarcoma, glioma, B-cell malignancies, oesophageal squamous cell carcinoma, and hepatocellular carcinoma |
| **MIR210HG** | −3.04 | / | / | −2.82 | −2.7 | / | Tumorigenicity | Glioma |
| **MIR22HG** | 5.91 | 2.84 | 0.92 | 3.07 | 4.33 | −1.35 | Tumorigenicity | Lung carcinoma |
| **MIR17HG** | 5.14 | 0.5 | / | 3.93 | 7.4 | / | Metastasis | Gastric and colorectal cancer |
| **SNHG1** | 0.69 | −0.8 | −2.23 | 8.52 | 10.46 | / | Proliferation, cell cycle, apoptosis | Hepatocellular carcinoma, neuroblastoma, prostate cancer |
| **SNHG5** | 0.98 | / | -0.52 | 0.66 | 3.93 | −0.52 | Proliferation, metastasis | Gastric cancer and melanoma |
| **SNHG12** | 5.97 | −0.73 | / | 3.85 | 3.96 | / | Proliferation, migration | Osteosarcoma |
| **HOTAIR** | −2.12 | / | / | / | −2.49 | / | Proliferation, migration, apoptosis, invasion, tumorigenicity | Gastric, breast, ovarian, bladder, cervical, colon, lung, colorectal, prostate cancer; gastrointestinal stromal tumor; pancreatic ductal adenocarcinoma; papillary thyroid carcinoma, glioma, hepatocellular carcinoma, osteosarcoma, esophageal squamous cell carcinoma; nasopharyngeal carcinoma, head and neck squamous cell carcinoma |
| **MALAT1** | 0.66 | / | / | / | / | / | Proliferation, invasion, migration, cell cycle, metastasis | Glioma, lung, colorectal, bladder, laryngeal, pancreatic, gastric cancer, hepatocellular carcinoma, and multiple myeloma |
| **LINC00152** | −0.63 | −0.63 | −0.55 | / | / | / | Proliferation, migration, apoptosis, invasion, cell cycle | Infantile hemangioma, colon cancer, gastric cancer, renal cell carcinoma, hepatocellular carcinoma |
| **H19** | 1.03 | / | / | 1.14 | / | / | Proliferation, migration, apoptosis, invasion, tumorigenicity, metastasis | Gastric, breast, lung, bladder, colorectal, ovarian and skin cancer, glioblastoma, renal cell carcinoma |

Note: AA indicates ACBP and ASLB combined treatment; CON indicates control MKN45 cells; / indicates not detectable.
